# Evaluation of Root Anatomy and Canal Configuration of Human Permanent Maxillary First Molar Using Cone-Beam Computed Tomography: A Systematic Review

**DOI:** 10.3390/ijerph191610160

**Published:** 2022-08-16

**Authors:** Sourabh Barbhai, Rajesh Shetty, Poonam Joshi, Vini Mehta, Ankita Mathur, Tanvi Sharma, Damini Chakraborty, Priyanka Porwal, Aida Meto, Dian Agustin Wahjuningrum, Alexander Maniangat Luke, Ajinkya M. Pawar

**Affiliations:** 1 Department of Conservative Dentistry & Endodontics, Dr. D.Y. Patil Dental College and Hospital, Dr. D.Y. Patil Vidyapeeth, Pimpri, Pune 411018, Maharashtra, India; 2 Department of Public Health Dentistry, Dr. D.Y. Patil Dental College and Hospital, Dr. D.Y. Patil Vidyapeeth, Pimpri, Pune 411018, Maharashtra, India; 3 STAT SENSE, Srushti 10, Sector 1 D, Amba Township Pvt. Ltd., Trimandir, Adalaj 382421, Gujarat, India; 4 Bhowal’s Dental and Implant Clinic, Bengal GreenField Heights, Galaxy-6FS, beside Apollo Pharmacy, DJ Block (Newtown), Newtown, Kolkata 700136, West Bengal, India; 5 Department of Dentistry, Faculty of Dental Sciences, University of Aldent, 1007 Tirana, Albania; 6 Department of Conservative Dentistry, Faculty of Dental Medicine, Universitas Airlingga, Surabaya 60132, East Java, Indonesia; 7 Department of Clinical Sciences, College of Dentistry, Ajman University, Ajman 346, United Arab Emirates; 8 Centre of Medical and Bio-allied Health Sciences Research, Ajman University, Ajman 346, United Arab Emirates; 9 Department of Conservative Dentistry and Endodontics, Nair Hospital Dental College, Mumbai 400008, Maharashtra, India

**Keywords:** canal configuration, root canal, Vertucci classification, permanent mandibular first molar, CBCT

## Abstract

The aim of this paper is to review the literature on root canal configuration (RCC) and the frequency of occurrence of a second mesiobuccal canal (MB) in human permanent maxillary first molars where cone-beam computed tomography (CBCT) is used. Online electronic databases such as PubMed-Medline, Embase, Scopus and Cochrane Library were searched using appropriate keywords from the earliest available date until 12th June 2022, without restriction on language. In the mesiobuccal root, type I was the most frequent (33.29%), followed by types II and IV (27.18% and 26.36%, respectively). Moreover, 68.2% of maxillary first molars had a second MB canal. For both the distobuccal and palatal roots, type I was the most prevalent, with 99.08% and 97.83% occurrence, respectively. All other types were infrequent. Type I RCC is most frequent in all the roots of the maxillary first molars. Hence, care must be taken during biomechanical preparation of the MB roots.

## 1. Introduction

Dental caries is among the most common chronic diseases [1]. If left untreated it can progress and infect the pulp and, subsequently, the periapical tissues, leading to irreversible pulpitis or apical periodontitis, respectively. The treatment of choice is root canal therapy. The main aim of this therapy is to remove bacteria and infected materials from the pulp and periapical tissues and replace them with biocompatible material [2,3,4]. According to Siqueira JF et al. and Lin LM et al., complex root anatomy is the primary cause of endodontic treatment failure [5,6]. Among various races, and different individuals within the same race, the morphology of the pulp canal varies momentously [7]. Thus, knowledge of the root canal’s configuration is essential for endodontic success [8].

A root may contain a simple canal that tapers and terminates into the apical foramen, or the configuration can be more complex, with multiple interconnecting canals, lateral branches and multiple foramina. Classifications of root canal configurations (RCCs) have been given by several authors. Weine was the first to classify canals present in one root into four types [9]. In 1984, Vertucci analyzed the canal anatomy and gave a classification with eight different types of canals [10]. Later, Sert and Bayirli added additional types to the Vertucci classification, giving a total of XXIII types of root canal configuration [11]. Recently, in 2017, Ahmed H et al. developed a new code system to classify root canals that also includes the number of roots present [12].

To navigate through these complex canal systems, proper radiographic aid is crucial. Radiography is essential in the diagnosis, treatment planning and success of endodontic therapy [13]. However, conventional radiographs only provide a two-dimensional view, resulting in the incomplete detection of root canals [14,15]. However, a detailed three-dimensional view of a tooth, along with its surrounding anatomical structure, is possible with the help of cone-beam computed tomography (CBCT) [16,17]. Blattner T et al. reported that CBCT acts as a much superior imaging method when compared with traditional radiographs in the diagnosis of second mesiobuccal canals [18]. In a study by Matherne et al. in 2008, it was found that while using digital radiographs, endodontists failed to detect at least one root in 40% of the tooth when compared with using CBCT [19]. Additionally, using CBCT as a methodology for in vivo studies aids in obtaining a greater number of samples, as it helps the analysis of full dentition of several patients collected from a specific population in a consecutive manner, thus allowing for adequate statistical analysis [20,21]. In human dentition, maxillary first molars are the second-most common teeth to undergo root canal treatment, immediately after the mandibular first molars [22]. Additionally, performing endodontic treatment of the mesiobuccal root of maxillary first molars is a challenge due to the significant prevalence of additional canals and morphological variations [23].

The main aim of this systemic review is to analyze the available studies on the prevalence of root canal configuration of maxillary first molar teeth assessed using CBCT to help dentists to successfully identify the root anatomy, and subsequently to perform endodontic treatment successfully.

## 2. Materials and Methods

This systematic review was conducted in accordance with the Preferred Reporting Items for Systematic Reviews and Meta-Analysis (PRISMA) statement guidelines [24]. The study protocol was registered and approved on the International Prospective Register of Systematic Reviews PROSPERO (Reg. No: CRD42021259436) before the start of the study.

### 2.1. Focused Question

What is the prevalence of root canal configuration and frequency of occurrence of a second mesiobuccal canal in the human permanent maxillary first molars where cone-beam computed tomography (CBCT) is used?

### 2.2. Inclusion Criteria

In vivo studies discussing the anatomy and canal configuration of permanent maxillary first molars were included. Only studies that used an in vivo CBCT methodology were included. The context included all of the in vivo studies carried out using CBCT, without excluding any country in the world. The population consisted of patients who had been subjected to CBCT, regardless of its diagnostic purposes. The primary outcome for this systematic review was to check the prevalence of root canal configurations of permanent maxillary first molars based on the Vertucci classification. 

### 2.3. Exclusion Criteria

- Studies using any classifications other than Vertucci.
- Case reports, case series and reviews were excluded.

### 2.4. Search Strategy and Data Collection

A literature search was performed in four major electronic databases—PubMed-MEDLINE, Cochrane Library, Embase and Scopus—along with additional sources, such as Google Scholar, major journals, unpublished studies, conference proceedings and cross references. A comprehensive search to identify studies related to root anatomy and the canal morphology of permanent maxillary first molar teeth was conducted until 12 June 2022, utilizing keywords such as “Vertucci classification”, “maxillary first molars”, “root anatomy” and “root canal configuration”. No additional filters or language restrictions were kept while conducting the searches. Two authors independently carried out the literature search, reviewed the study articles and extracted data. The screening was performed in two stages. First, the titles and abstracts of all of the articles were reviewed, followed by full text screening. Those studies that fulfilled the selection criteria were processed for data extraction. Non-English language publications were translated into the English language using Google Translate [25]. The information was independently extracted by the two authors using specially-designed data-extraction forms utilizing Microsoft Excel software. Any disagreement was resolved by discussion between the authors. For each selected study, the following data were then extracted from a standard form (when available): author and year of publication, sample size, population, root number, root canal configuration, CBCT model and CBCT settings.

### 2.5. Quality Assessment

The checklist given by Martins JNR et al. in Preferred Reporting Items for Epidemiologic Cross-sectional Studies on Root and Root Canal Anatomy Using Cone-beam Computed Tomographic Technology was used for quality assessment [26]. The quality of the included articles was evaluated across 6 domains: title, keywords, aim, methodology, results and discussion. Two authors individually evaluated each topic in the abovementioned domains and gave a score of 1 (reported) or 0 (not reported) for each of the included articles.

## 3. Results

### 3.1. Search Selection and Results

**Figure 1:**
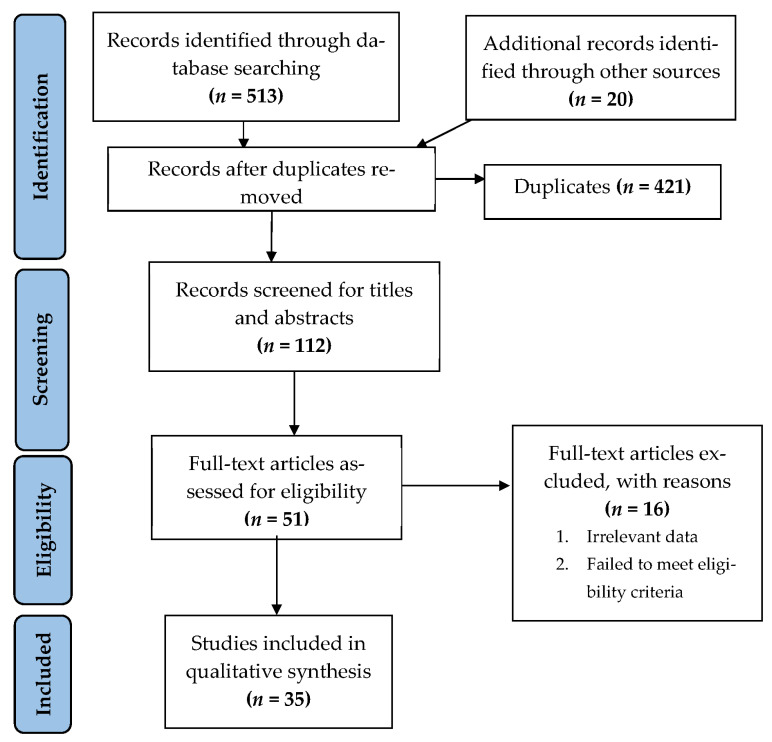
Flowchart summarizing the article selection process (*n*—number of studies).

After extensive searching, a total of 533 studies were identified, out of which 421 were duplicates. The remaining 112 studies underwent title and abstract screening, and 51 studies were selected for full text screening. Sixteen studies were excluded after full text screening. Thus, a total of 35 studies that met our inclusion criteria were processed for data extraction [27,28,29,30,31,32,33,34,35,36,37,38,39,40,41,42,43,44,45,46,47,48,49,50,51,52,53,54,55,56,57,58,59,60,61] (Figure 1).

### 3.2. Study Characteristics

The 35 included studies were conducted across the globe, with wide demographic variations, and a total of 17278 permanent maxillary first molar teeth were identified. Nearly all the studies were conducted on adult populations, except for two [45,48]. One study was based on variations in RCC among various age groups, and one of the selected age groups was younger than twenty years [45]. The second study was conducted among children of age groups ranging between 9 and 12 years. All studies provided details of CBCT specifications except for the study by Raja M et al., where details about the CBCT were not available [51]. The details of CBCT software, setting, field of view (FOV), voxel size and visualization software are shown in Table 1.

### 3.3. Outcome

In total, 35 studies presented data on the canal configuration of maxillary first molars based on the Vertucci classification [27,28,29,30,31,32,33,34,35,36,37,38,39,40,41,42,43,44,45,46,47,48,49,50,51,52,53,54,55,56,57,58,59,60,61]. The concurred data for most of these studies included both the percentage of occurrence and the number of cases. For a few studies, only the percentage of occurrence was given, and the exact value of the number of teeth for each specific canal type was calculated from the given percentage and sample size (number of teeth) taken for the study. To draw a definite conclusion among the myriad of data extracted from the studies and to interpret the data properly, each type’s total percentage was calculated for the mesiobuccal (MB), second mesiobuccal (MB2), distobuccal (DB) and palatal (P) canals separately.

### 3.4. Prevalence of Canal Configuration of Mesiobuccal Root Based on Vertucci Classification

Different studies, 31 of them, have reported the root canal configurations of the mesiobuccal root of the maxillary first molar [27,28,29,30,31,33,34,35,36,37,38,39,40,41,42,43,44,45,46,47,48,49,51,52,53,54,55,56,58,59,61]. The data from the studies were pooled to find the mean of all eight types of canal configurations based on Vertucci classification. Among them, type I was the most frequent, with 33.29%, followed by types II and IV with 27.18% and 26.36%, respectively.

### 3.5. Prevalence of Canal Configuration of the Second Mesiobuccal Root Based on Vertucci Classification

Only four studies reported the canal configuration of the second mesiobuccal root. Type II was seen to be the most frequent with 37.4%, followed by type IV and type I with 22.9% and 20.3%, respectively [32,50,57,60].

### 3.6. Prevalence of Canal Configuration of the Distobuccal Root Based on Vertucci Classification

Another 23 studies reported the canal configuration of the distobuccal root of the maxillary first molar [28,29,30,31,33,34,35,36,38,40,41,44,45,46,47,48,49,51,52,53,55,56,61]. Out of these, type I was the most prevalent, with a range from 97.83% to 99.08% occurrence.

### 3.7. Frequency of Occurrence of Second Mesiobuccal Canal

Additionally, 25 studies reported the presence of a second mesiobuccal canal. Out of the 12056 teeth, a total of 8223 teeth showed the occurrence of MB2 canals, i.e., 68.2% occurrence [27,28,29,30,32,35,36,37,39,40,41,42,43,44,46,47,48,50,51,52,53,54,55,56,57] (Table 2).

### 3.8. Quality Assessment

All included studies reported the following domains: aim of the study, morphology concept, assessment methodology, sample size and generalizability of the outcomes. Future research was the least reported domain, followed by the strengths and limitations of the study design. In the title, all studies mentioned CBCT but failed to indicate the type of study being conducted, except for one study by Kalender et al. which mentioned both [33] (Table 3).

## 4. Discussion

Before discussing the results, it must be noted that all the studies had variable CBCT settings and specifications. This, along with the demographic variations in the samples across the studies, will have some influence on the results. Among the studies included for this systematic review, three rooted maxillary first molars were most commonly reported. A similar finding was reported by Peris R et al. [62]. Some studies reported the presence of roots as being one, two and four, although these were infrequent [28,29,35,41,44,48]. Only three studies reported the RCC of maxillary molars with four roots, while only one study by Tian et al. in a Chinese population reported the RCC of the maxillary first molar with only one root [35,44,48]. Al-Shehri S et al. also reported the presence of fused roots along with RCC among maxillary first molars [41].

The main outcome of this systematic review was to determine the prevalence of RCC among maxillary first molars. Most studies reported a higher prevalence of Vertucci types I, II and IV in the mesiobuccal root. After pooling the data from all the studies, type I was observed as the most prevalent type in MB roots, with 33.29% occurrence. Types II and IV had similar prevalence rates of 27.18% and 26.36%, respectively. Our findings are similar to those of other studies, which show type I to be the most prevalent, followed by types II and IV [63,64]. In a comparative study by Peris R et al. on Sri Lankan and Japanese populations, the same trend was seen for the Japanese population, but for Sri Lanka, the second most common was type V [62]. In two studies, type V was reported to have a higher frequency of occurrence [27,42]. Both of these studies were conducted on the Chinese population. In the study by Zang et al., among 299 tooth samples, 70% had type V, making it the most prevalent RCC type [27]. In the rest of the studies, other RCC types were infrequent and had a very low percentage of occurrence [28,29,30,31,32,33,34,35,36,37,38,39,40,41,43,44,45,46,47,48,49,50,51,52,53,54,55,56,57,58,59,60,61]. Among 15196 teeth samples, only 0.36% of mesiobuccal roots were reported to have root canal configurations outside of the Vertucci classification, and only 9 studies out of 35 reported this finding [28,33,35,41,46,48,49,53]. In particular, four studies reported the root canal configuration of the second mesiobuccal canal [32,50,51,60]. All of them reported type II as the most frequently occurring RCC, followed by type III and type I. Among the four studies, a sample size of 2019 teeth, was present, of which 37.4% were type II [32,50,51,60]. None of the studies reported findings for type VIII. 

The root canal configuration of the distobuccal and palatal roots was less complex. Both of these roots mostly had a single root canal. All studies, with no exception, reported type I as the most frequent root canal configuration in both the distobuccal and palatal roots. All other types were infrequent. Among the 11660 tooth samples, 97.83% of palatal roots and 99.08% of distobuccal roots had type I RCC. Thus, in all three roots, mesiobuccal, distobuccal and palatal, type I was the most prevalent root canal configuration in maxillary first molars. This finding is similar to three other studies that highlight a higher prevalence of type I and a very low frequency of occurrence in all other types in both distobuccal and palatal roots [61,65,66,67].

Twenty-five authors acknowledged the presence of an additional mesiobuccal canal, and most of them reported a higher prevalence of MB2 canals [27,28,29,30,32,35,36,37,39,40,41,42,43,44,46,47,48,50,51,52,53,54,55,56,57]. In the study by Soh et al. on the Indian population, the frequency of occurrence of MB2 canals was the lowest, at only 30% [55]. Alsaket YM et al. in 2020 reported a maximum frequency of MB2 canals of 87% in their study on the population of Jordan [57]. The mean percentage of MB2 canals was 68.2%. Faraj BM in 2021 concluded that the MB2 canal was found in 53.78% of the teeth. In a study performed by Martins et al. looking at the worldwide prevalence of MB2 canals using CBCT, the overall prevalence was 73.8% [68]. Bentancourt P et al. found 69.82% of MB2 canals in their study on 1100 maxillary molars using CBCT [69]. Even though the CBCT specifications changed across the studies, the Newtom CBCT scanner was the most commonly used scanner. All the studies had a similar methodology for the assessment of the CBCT scans. Experienced endodontists or radiologists viewed the CBCT in 3 planes: axial, coronal and sagittal. For the identification of root anatomy, CBCT is a much better diagnostic tool than periapical radiography [70]. Abuabara A et al. reported that periapical radiographs can detect only 8% of MB2 canals, while with the help of CBCT, a second mesiobuccal canal can be detected in 54% of teeth [71]. Maxillary molars with 2 canals are frequently misdiagnosed, and 78.4% of MB2 canals remain unfilled [72]. Due to the higher presence of unexpected root canals in the maxillary mesiobuccal root, the chances of root canal treatment failure are higher [72,73]. However, in the distobuccal and palatal roots, the anatomy was simple. Type I RCC was highly prevalent, and the number of canals was mostly limited to one per root. Thus, the chances of missing a root canal or failed root canal therapy are lower. In this systematic review, we found that the mesiobuccal roots most commonly have type I RCC, followed by types II and IV. Gaêta-Araujo H et al. found that most teeth without endodontic technical errors had type I RCC [74]. If technical errors are present with type I, they are due to underfilling or nonhomogenous filling [75,76]. 

The sample sizes (number of teeth) among the studies varied. Thus, the percentages of studies with a small sample size (number of teeth) were higher, even though we tried to obtain a conclusive finding by calculating the percentages. Hence, pooling the data to find an overall mean percentage helped us to achieve a more conclusive result. However, our study has certain limitations. The inclusion criteria only helped to establish homogeneity among the methodologies used in various studies. However, the data collected had samples of patients from all age groups, genders and from different parts of the world, with varied ethnicities and genetic predispositions. These factors might have influenced the findings. Additionally, even though only studies using CBCT as a methodology were included, the CBCT parameters and specifications across all of the studies were not the same. In future studies, a more selective CBCT specification and data pooling based on ethnicities can be conducted to obtain more homogeneous results. The use of a limited-view CBCT device with specified resolution and lower voxel size will provide superior image quality, helping to explore the root and canal morphology more accurately. Root canal systems of maxillary first molars are complex and unpredictable. They vary among populations, and even in individuals in the same population. Cohort studies, in which the same individuals are observed over time, are necessary to analyze and describe various factors, such as age, which can determine whether MB canals narrow or calcify in a canal, and whether age can affect the number and size of the MB canals in maxillary molars. 

## 5. Conclusions

From this systematic review, we can conclude that type I RCC is most frequent, based on the Vertucci classification of the maxillary first molars. Palatal and distobuccal roots have a more-or-less simple anatomy, with one canal and mostly type I configuration. However, the mesiobuccal root has a more complex anatomy due to the high frequency of occurrence of a second mesiobuccal canal; furthermore, in the mesiobuccal root, the occurrence of type II RCC, which is closely followed by type IV RCC, is more common. Hence, care must be taken during biomechanical preparation of the mesiobuccal roots. CBCT can act as an auxiliary to help endodontists obtain a better visualization of the anatomy of the mesiobuccal root and help in detecting additional canals, thus ensuring successful endodontic treatment.

## Figures and Tables

**Table 1:** Cone-beam computed tomography parameter values of each study.

Study/Year of Publication	Country	CBCT Model	Voxel Size	FOV	Settings CBCT	Software Visualization
Zang R et al./2011 [27]	China	3D Accuitomo scanner (Morita, Kyoto, Japan)	0.125 mm	40 mm or 60 mm	80 kV and 5.0 mA, time 17 s	i-Dixel one volume viewer 1.5.0 and a Dell Precision T5400 workstation (Dell, Round Rock, TX, USA)
Kim Y et al./2012 [28]	Korea	Dinnova system (Willmed, Gwangmyeong, Korea)	0.167-mm^3^	10 cm	80 kVp, 9.0 mA	OnDemand3D software (Cybermed, Seoul, Korea).
Tocci L et al./2013 [29]	Italy	NewTom VGi Vertical Cone Beam (Verona, Italia)	0.3 mm	15 cm	110 Kvp, 1–20 mAs, 15 mSv	NA
Guo J et al./2014 [30]	USA	Sirona Galileos device (Sirona Dental Systems, Inc, Long Island City, NY, USA)	0.3/0.15 mm.	15 cm	85 kV and 5–7 mA	The Digital Imaging and Communications in Medicine (DICOM) format images were exported from Galileos and imported into InVivo Dental Application 5.1.6 software (Anatomage Inc., San Jose, CA, USA).
Altunsoy M et al./2014 [31]	Turkey	CBCT scanner (ICAT Vision; Imaging Science International, Hatfield, PA, USA)	0.3 mm	NA	120 kVp, and 18.54 mA, 8 s	NA
Abarca J. et al./2015 [32]	Chile	Gendex CB500 imaging system	0.2 mm	NA	120 kVp and 5 mA and 0.2 mm thickness of the cut	iCATVision software v 1.8.1.10 in a darkroom on 21” LCD monitors with a resolution of 1280 × 1024 pixels.
Kalender A et al./2015 [33]	Turkey	Newtom 3G: Quantitative Radiology s.r.l., Verona, Italy	NA	9 inch	NA	NNT 4.6, QR Verona, Italy
Naseri M et al./2016 [34]	Iran	NewTom VGi (QR SRL Company, Verona, Italy)	200 µm	8 × 12 cm	110 kVp and exposure time of 3.6 s	NewTom NNT software version 5.3 (Quantitative Radiology, Verona, Italy)
Tian X et al./2016 [35]	Chinese	NewTom VG; QR srl, Verona, Italy	0.16 mm	500 cm^2^ (20 × 25 cm)	110 kVp and 10 mA, 18 s	NNT software version 2.21 (ImageWorks, Elmsford, NY, USA)
Martins J.N.R et al./2016(I) [36]	Portugal	Planmeca scanner (Planmeca Promax, Planmeca, Finland)	0.2 mm	NA	80 kv, 15 mA, 12 s	Planmeca Romexis, Planmeca
Al-Kadhim A et al./2017 [37]	Malaysia	NA	NA	NA	NA	One Data Viewer software (J. Morita Manufacturing Corp).
Perez M et al./2017 [38]	Spain	9300 3D CBCT unit (Carestream Dental, Atlanta, GA, USA)	0.18 mm	10 × 10 cm	90 kV, 4 mA, 8 s	Carestream software (CS 3D Imaging software 6.1.4)
Zand V et al./2017 [39]	Iran	NewTom GI CBCT (Verona/Italy)	NA	NA	110 kVp, 18 s	NNT viewer software program
Ghobasby A et al./2017 [40]	Egypt	Cranex 3D (Soredex ,Tuusula, Finland)	133-μm	NA	80 kVp, 9.0 mA	NA
Al-Shehri S et al./2017 [41]	Saudi Arabia	1. I-CAT (Imaging Science International, Hatfield, PA, USA), 2. Galileos (Sirona Dental Systems, Bensheim, Germany), 3.Carestream CS 9300 (Carestream Health, Inc., Rochester, NY, USA).	0.3 mm (14-bit grayscale)	NA	85 kV, 5–7 mA	OnDemand3D software (Cybermed, Seoul, Korea)
Wang H et al./2017 [42]	China	Planmeca Romexis 3D CBCT scanner (Planmeca, Finland)	200 μm	NA	84 kV and 14 mA,12 s, the minimum slice thickness was 0.2 mm.	The CBCT images were 3D-reconstructed by using a patented Feldkamp reconstruction algorithm, analysed with inbuilt software and ran in a 32-bit Windows 7 system.
Khademi A et al./2017 [43]	Iran	Galileos (Sirona Dental Systems Inc., Bensheim, Germany)	150 μm	150 × 150 or 75 × 150 mm	85 kVp, 42 mA	SIDEXIS XG software version 3.7 (Sirona Dental System GmbH, Bensheim, Germany).
Ghoncheh Z/2017 [44]	Iran	NewTom VG CBCT system (Image Works, Verona, Italy)	0.3 mm	(11 × 16 cm	110 kV, 1–20 mA, 3.6–5.4 s.	NNT Viewer software (NNT 2.21; Image Works, Verona, Italy).
Martins J.N.R et al./2018(II) [45]	Portugal	Planmeca Promax, Planmeca, Helsinki, Finland	0.20 mm	NA	80 kV, 15 mA, 12 s	Romexis visualization software (Planmeca)
Martins J.N.R et al./2018(III) [46]	China	Kodak 9500	0.2 mm	Full Arch	90 kV, 10 mA, 10.8 s	CS 900 3D imaging
	Portugal	Planmeca Promax	0.2 mm	Full Arch	80 kV,15 mA, 12 s	Planmeca Romexis
Razmuvo S et al./2018 [47]	Moscow	3D eXam (KaVo, Biberach, Germany)	0.3 mm	23 cm × 17 cm	110 kV, 1.6–20 s	g I-CAT viewer software (version 10, Hatfield, England).
Ratanajirasut et al./2018 [48]	Thai	3D Accuitomo CBCT machine (J Morita Manufacturing Corp, Kyoto, Japan	0.25 mm × 0.25 mm	100.025 × 100.025	80 kVp,5 mA, 17.5 s	g One Volume Viewer software (J Morita Manufacturing Corp)
Martins J.N.R et al./2018(IV) [49]	Portugal	Planmeca Promax	0.2 mm		80 kV,15 mA, 12 s	Planmeca Romexis
Alves CRG et al./2018 [50]	Brazil	Prexion 3D Elite model XP68 (PreXion Inc., San Mateo, California, USA),	0.15 mm (for FOV 8) and 0.11 mm (for FOV 5)	5 [5.6 cm × 5.2 cm (partial jaw) ] or 8 [8.1 cm × 7.5 cm (total jaw)]	90 Kvp and 4 mA, 37 s	3D software PreXion Image Analysis System (PreXion Inc. San Mateo, California, USA)
Raja M et al./2018 [51]	India	NA	NA	NA	The CBCT scanner was set at a constant slice thickness of 125 μm/slice	NA
Pan YJ et al./2019 [52]	Malaysia	KaVo 3D eXam imaging system (Imaging Sciences International, Hatfield, PA, USA).	0.25 mm	NA	121 kVp, 5 mA, 26.9 s	eXam Vision software version 1.9.3.13 (KaVo Dental GmbH, Biberach, Germany)
Mohara NT et al./2019 [53]	Brazil	a 3D Accuitomo 80 CBCT (J. Morita, Kyoto, Japan)	NA	40 mm or 60 mm	90 KVA, 8 mA, 18 s	i-Dixel (J Morita, Tokyo, Japan)
Candeiro GTM et al./2019 [54]	Brazil	Prexion 3D imaging device (Prexion, Inc., San Mateo, USA)	0.125 mm	NA	90 kVp and 4 mA	(Prexion, Inc., San Mateo, USA) was used on a Dell Precision T5400 (Dell, Round Rock, TX, USA)
Soh N et al./2019 [55]	India	NA				
Al Mheiri E et al./2019 [56]	United Arab Emirates	Planmeca ProMax CBCT scanner (Planmeca Oy, Helsinki, Finland)	0.4 mm	16 × 11 cm	120 kVp, 18.54 mA, 8.9 s	iMAC computer ([27-in. screen size with Retina 5 K display, 5120 × 2880 resolution with support for 1 billion colors, 500 nits brightness], Apple, USA) in a room with controlled lighting using the Horos DICOM viewer
Alsaket YM et al./2020 [57]	Jordan	Carestream Dental, Rochester, NY, USA	NA	NA	NA	NA
Liu Y et al./2020 [58]	China	NewTom VG scanner (QR srl, Verona, Italy)	0.125 mm	Small	NA	3D reconstructed with an open source software platform 3D Slicer 4.8.1 from Slicer web site
Popovic M et al./2020 [59]	Serbia	Orthophos XG 3D device (Sirona Dental Systems GmbH, Bensheim, Germany)	160 μm	0.16 mm	NA	GALAXIS v1.9.4 (Sirona Dental Systems GmbH, Bensheim, Germany)
Al-Saedi A et al./2020 [60]	Iraq	Gendex (GXDP-7000) CBCT machine (Hatfield, PA, USA)	200 µm	80.0 × 80.0 × 60.0 mm	90 kV,10 mA, 13 s	Software GxPicture; Kavo Dental, Biberach a der Riss, Germany built into the Invivo 5 dental viewer (Anatomage, San Jose, CA, USA) and run on a 64-bit Windows 7 system (Microsoft Corporation, Redmond, WA, USA)
Nikkerdar N et al./2020 [61]	Iran	New Tom VGi CBCT system (QR SRL Co., Verona, Italy)	0.15 mm	120 × 80 mm	110 kVp, 10 mA, 5.4 s	NNT Viewer version 7.2 software on a 12.5-inch laptop (Asus) with 1080 × 1920 p resolution

CBCT: cone beam computed tomography, FOV: field of view, mm: millimeter, µm: micrometer, kVp: kilovoltage peak mA: milliamper, s: seconds, mSv: millisievert.

**Table 2:** Prevalence of MB2 canals in maxillary first molars.

Study/Year of Publication	Sample Size (*n*)	Population	MB2 Canals *n* (%)
Zang R et al./2011 [27]	299	China	155 (52%)
Kim Y et al./2012 [28]	814	Korea	510 (62.65%)
Tocci L et al./2013 [29]	161	Italy	62 (40.3%)
Guo J et al./2014 [30]	628	USA	428 (68.2%)
Abarca J. et al./2015 [32]	802	Chile	802 (73.44%)
Tian X et al./2016 [35]	1536	China	820 (53.9%)
Martins J.N.R et al./2016 (I) [36]	421	Malaysia	191 (45.6%)
Al-Kadhim A et al./2017 [37]	494	Portugal	350 (71.05%)
Zand V et al./2017 [39]	156	Iran	86 (55.11%)
Ghobasby A et al./2017 [40]	605	Egypt	451 (74.5%)
Al-Shehri S et al./2017 [41]	330	Saudi Arabia	195 (55.6%)
Wang H et al./2017 [42]	939	China	641 (68.3%)
Khademi A et al./2017 [43]	389	Iran	272 (70.2%)
Ghoncheh Z/2017 [44]	337	Iran	155 (46%)
Martins J.N.R et al./2018 (III) [46]	239	China	552 (67.35%)
Razmuvo S et al./2018 [47]	410	Moscow	382 (59.8%)
Ratanajirasut et al./2018 [48]	476	Thai	303 (63.6%)
Alves CRG et al./2018 [50]	362	Brazil	247 (68.23%)
Raja M et al./2018 [51]	500	Indian	400 (80%)
Pan YJ et al./2019 [52]	344	Malaysia	125 (36.3%)
Mohara NT et al./2019 [53]	326	Brazil	209 (64.22%)
Candeiro GTM et al./2019 [54]	700	Brazil	337 (48.21%)
Soh N et al./2019 [55]	66	India	20 (30%)
Al Mheiri E et al./2019 [56]	522	United Arab Emirates	418 (80.1%)
Alsaket YM et al./2020 [57]	200	Jordan	174 (87%)
Total	12,056		8223 (68.2%)

**Table 3:** Specific preferred reporting items for cross-sectional studies on root and root canal anatomy using cone-beam computed tomography (CBCT).

Sr. No.	Section Item	Total (*n*)	Percentage (%)
1	Title	33	94.29
**Introduction**			
2	Keywords	32	91.43
3	Aim	35	100.00
**Methods**			
4	Participants (in vivo assessment)	33	94.29
5	CBCT	33	94.29
6	Morphology concept & assessed teeth (variables)	35	100.00
7	Assessment	35	100.00
8	Observers	27	77.14
9	Potential sources of bias	21	60.00
10	Final sample size	35	100.00
11	Reliability	25	71.43
12	Statistical analysis	33	94.29
13	Ethics committee Results	24	68.57
14	Primary Outcomes	34	97.14
15	Other analysis	28	80.00
16	Visual documentation Support	31	88.57
**Discussion**			
17	Outcome interpretation	35	100.00
18	Strength & limitations	23	65.71
19	Generalizability	35	100.00
20	Future research	5	14.29

## Data Availability

The reported systematic review and meta-analysis is registered with PROSPERO reg no.: CRD42021259436 available at https://www.crd.york.ac.uk/prospero/export_details_pdf.php (accessed on 13 August 2022).
